# Prevalence of malnutrition & anemia in preschool children; a single center study

**DOI:** 10.1186/s13052-023-01476-x

**Published:** 2023-06-16

**Authors:** Hanan Mina Fouad, Aly Yousef, Ahmed Afifi, Ahmed A. Ghandour, Amira Elshahawy, Ayman Elkhawass, Hazem Hawees, Eman Shaheen, Mohamed Alaaeldin, Mostafa Kamal, Samah Bastawy, Samah Rabie, Farah Wissa, Sherine Shalaby

**Affiliations:** 1 Pediatrics Department, Helwan University, Cairo, Egypt; 2 National Hepatology and Tropical Medicine Research Institute (NHTMRI), Cairo, Egypt; 3 Community, Environmental and Occupational Medicine Department, Helwan University, Cairo, Egypt; 4 Pediatric Department, the National Nutrition Institute, General Organization of Teaching Hospitals, Ministry of Health, Cairo, Egypt; 5 Intern in Helwan University Hospital, Cairo, Egypt; 6 Clinical and chemical Pathology Department, Helwan University, Cairo, Egypt; 7 Psychiatry Department, Helwan University, Cairo, Egypt; 8 Pediatrics Department, Suez Canal University, Ismailia, Egypt

**Keywords:** Anemia, Anthropometry, Overweight/obese, Preschool age, Stunting, Undernutrition, Wasting

## Abstract

**Background:**

Malnutrition including undernutrition, overnutrition, and micronutrient deficiencies are considerable problems worldwide, with variable burdens among different communities. Its complications include physical and cognitive impairment, with the probability of irreversible lifelong consequences. We aimed to assess the prevalence of undernutrition, overweight, obesity, and anemia in preschoolers, being a risk group for developmental adverse events.

**Methods:**

We recruited 505 healthy preschool children, with a male: female ratio of 1.05:1. Children with chronic diseases were excluded. We used anthropometry and complete blood count to screen for malnutrition and anemia.

**Results:**

The mean age of the study group was 3.8 ± 1.4 years (1.02–7). The screening results were average in 228 (45.1%) children, while 277 (54.9%) children had either abnormal anthropometry, anemia, or both. We observed undernutrition in 48 (9.5%) children; among them, 33 (6.6%) were underweight, 33 (6.6%) wasted, and 15 (3%) were stunted, with no significant difference between children aged below or above five. We identified overnutrition in 125 (24.8%); 43 (8.5%) were overweight, 12 (2.4%) were obese, and 70 (13.9%) had a high body mass index Z score, not qualifying the definition of overweight. Anemia was diagnosed in 141 (27.9%) children and was significantly more frequent among older children without gender discrimination. About 10% (50 children) had both anemia and abnormal anthropometry. The frequency of abnormal anthropometry was comparable between children with anemia and those with normal hemoglobin.

**Conclusion:**

Malnutrition and anemia in preschoolers are still a heavy burden affecting about half of our study group, with an upward trend towards the overnutrition side. Anemia is still a moderate public health problem in preschoolers.

## What’s known on this subject

Malnutrition in preschoolers is a considerable problem worldwide, with variable burdens among different communities. The current situation of these problems requires a precise estimation to cope with the WHO's call to reduce the prevalence of malnutrition and anemia in children.

## What this study adds

Malnutrition and anemia in preschoolers are still a heavy burden affecting about half of our study group, with a shift of the weight of malnutrition towards the overnutrition side. As a national observation, an upward trend for overweight & obesity was associated with a declining one for undernutrition. Anemia is still a moderate public health problem in preschoolers.

## Background

Children struggle with multiple health hazards affecting their current and future health status. A significant burden is owed to non-communicable diseases induced by an imbalance in nutritional intake, i.e., diet-related. Being widely prevalent, malnutrition in children represents an important cause of morbidity and mortality. It comprises various presentations, including undernutrition, micronutrient deficiencies due to inadequate vitamins or minerals, and obesity. Unfortunately, malnutrition's global burden is severe and long-standing, with medical, economic, social, and developmental impacts affecting individuals and their families, communities, and countries [1].

One form of malnutrition is undernutrition; its current rates reach 50% in the Eastern Mediterranean Region [2] and are associated with long-term sequelae and mortality among affected children. It causes about half of deaths in children below five years in low- and middle-income countries [1].

Anemia is a prevalent finding in children; it affects about 42% of preschool children worldwide. Anemia caused by iron deficiency is the most prevalent type in developing and developed countries; it is a common form of micronutrient deficiency. As a rough estimation, up to 50% of anemias are caused by iron deficiency [3], with variable frequencies between communities [4]. Predisposing factors in children included living in low socioeconomic communities, the high rate of anemia in pregnant females (~ 40%), lack of awareness regarding proper nutrition in infancy/childhood, unhealthy feeding habits, and lack of regular follow-up assessment visits. Anemia at a young age concerns the evolving central nervous system maturation. When anemia is untreated at an early age, it can negatively affect many vital systems, e.g., the cardiovascular system), cognitive functions and learning abilities, and possibly long-lasting sequelae [1].

The general pattern of children's growth has changed over the last decades, even in developing countries, with augmented overweight/obesity rates and comparatively lower stunting rates. This nutrition transition has occurred due to consumption of diet rich in fat in association with less physical activity in daily life. The rates of overweight/obesity in children below five years increased by about 24% in Africa in the last decade, and currently, about 50% of Asian preschoolers are overweight or obese [1]. Prevalent complications during adolescence and early adulthood accompany the high rates of obesity in children, which are primarily irreversible, including cardiovascular diseases, e.g., heart diseases, hypertension, certain cancers, and Diabetes Mellitus type 2 [5]***.***

In our work, we aimed to estimate the prevalence of underweight, overweight/obesity, and iron deficiency anemia in apparently healthy preschool children in Egypt.

## Methodology

Our protocol was approved by the faculty of Medicine, Helwan University-research ethics committee before conduction, and we obtained written broad consent from one of the parents for each child. The screening was done by a cross-sectional design at Helwan University hospital in Badr city from November 2021 to June 2022.

We recruited asymptomatic preschool children who fulfill the following criteria: Egyptian children of both genders, aged between 2–7 years (7 is the average age for national school entry). Children known to have anemias for causes other than iron deficiency or diagnosed with any systemic illness causing abnormal growth and those with secondary obesity were excluded from the study.

### Study procedure

- *Project Announcement*: We encouraged parents living in the geographical area served by the faculty hospital to bring their healthy preschool children. We designed invitations including the project's aim, the weekly working day's date and time, participation benefits, and contact for inquiry. We sent invitations using social media, e.g., the hospital's official website and social groups in the city. In addition, we designed a Google form, and its link was added to the invitations for literate parents to register their children. We also used posters at target points in the hospital, namely the entrance and the pediatrics clinic.

- *Screening phase: *a campaign was conducted on a fixed pre-announced weekly day at the hospital when the other outpatient activities were absent to provide enough space without overcrowding. First, the planned project activities were presented to the parents in the waiting area to ensure their cooperation and acceptance of the time spent. Secondly, children and their parents went through separate stations for registration, anthropometric assessment, medical and dietetic History taking, detailed clinical examination, and nutrition awareness session. Finally, a blood sample was drawn for laboratory testing. Parents were requested to come again on the next working day of the project to obtain the assessment result, a copy of the laboratory results, and a follow-up card when required.
  - ◦ *Demographic data:* The children's age was calculated from the date of birth on their birth certificate and date of measurement. Also, the gender, residence, and contact information were documented.
  - ◦ *History taking*: emphasizing the medical History of the perinatal period, breastfeeding details, weaning, vaccination, developmental milestones, birth measures when available, physical activity, and sleep characteristics. We also reported the History of the current nutritional intake.
  - ◦ *Anthropometric assessment: *Two trained team members helped each other. The measurer held the child and took the measurements, and the other assisted with the process and recorded the measurements on the documentary sheet. We used uninflated colorful balloons to calm the children. *Weight and height* were measured using a stadiometer while the child was barefoot with minimum clothing. The weights of frightened children were measured by subtracting the mother's weight holding her infant in her arms from the total weight. Height was measured while the child looked straight ahead (at his mother who stood in front of him), the child's line of sight parallel with the ground, and the measurer's left hand under the child's chin, his shoulders at the same level, and his hands at his sides. Ensuring that the head, shoulder blades, and buttocks were against the measuring scale, the measurer lowered the headpiece on the child's head with his right hand. After that, BMI was calculated by dividing the weight measured in kg by the height in squared meters. Measures were plotted using the CDC growth charts for ages 2–20. Children with weight below the 3rd percentile or BMI below the 5th percentile for age & sex were labeled with undernutrition, and then classified to stunting or wasting as mentioned below. Children having BMI between 85th & 95th percentiles were considered overweight & those above 95th were considered obese [5]. Children with a height below the 3rd percentile for age & sex, and not underweight were labeled as short stature, with no further discussion being not our study aim. During data analysis, the Z scores for anthropometric measures were calculated online [6]*,* and cases were categorized accordingly to the international classification of malnutrition.
  - ◦ *Clinical examination: particularly concerned* about vitamin deficiency signs, systemic affection, and complications in children with obesity. The temperature was measured before entry, and febrile children were deferred for another appointment. Blood pressure was measured for cases of obesity using a sphygmomanometer with a suitable-sized cuff.
  - ◦ *Nutrition awareness:* flyers with details about healthy diet for preschoolers were distributed.
  - ◦ *Laboratory testing:* A 3 ml blood sample was obtained for all screened children to perform a complete blood count. In cases with anemia, we measured serum ferritin levels and CRP to assess the infection status.

## Definitions applied

- 1. Undernutrition: for children up to 19 years [7]:
  **Table:** 

**Undernutrition**	**Underweight**	**Stunting**	**Wasting/ Thinness**^**a**^
***Indicators specific to a child’s gender***	Weight-for-age	Height-for-age	Weight-for-height or BMI for age
***Z score classification:***			
*- Possible abnormality*	> + 1	> + 3	< + 2 to > + 1^b^
*- Normal*	< + 1 to ≥ -2	< + 3 to ≥ -2	< + 1 to ≥ -2
*- Moderate*	< -2 to ≥ -3	< -2 to ≥ -3	< -2 to ≥ -3
*- Severe*	Below > -3	Below > -3	Below > -3

^a^The term "wasting" is used for children aged up to 60 months, while thinness is for those aged between 5 and 19 years

^b^This range defines overweight in children aged between 5 to 19 years or at risk of being overweight in those under five years
-
  - *Stunting*: Indicates that a child does not reach their required growth potential. It reflects chronic malnutrition for over three months, affecting the height velocity [8] *.* It could be moderate or severe.
  - *Wasting*: Indicates that a child is skinny as regards their height. It is one of the indicators of acute malnutrition in children. It is either moderate or severe. *Severe wasting* can also be described as nutritional marasmus.
  - *Underweight*: Indicates that a child's weight is less than expected for a well-nourished, healthy child of the same age and gender. It indicates stunting or wasting without precise differentiation. It reflects either weight loss or inappropriate weight gain.

- 2. *Overweight and obesity*: for similar height and gender, overweight usually reflects excess weight from muscle, bone, fat, water, or a combination of them, while obesity usually reflects excess body fat [7]:

**Table:** 

**Overweight /Obesity**		
***Age***	Up to 5 years	5 to 19 years
***Indicator:***	Weight-for-height	BMI
***Z score classification:***		
*Overweight*	> + 2 to ≤ + 3	< + 2 to > + 1
*Obesity*	> + 3	> + *2*

- 3. *Anemia*: was defined when the hemoglobin level is below 11 gm% up to the age of 4 and 11.5% for ages 4–6 years [9]. Iron deficiency anemia was suspected when the mean corpuscular volume “MCV” was < 70 μm^3^, and red diameter width “RDW” > 15.

### Statistical analysis

Data was analyzed using IBM® SPSS (Statistical Package for Social Science) ® Statistics version 29. The participants' numerical data were presented according to the homogeneity of data distribution as mean ± standard deviation (SD) or median (interquartile range-IQR). We used the independent Student's t-test, or One-Way ANOVA, to test the difference between groups. Qualitative data were presented as frequency and percentage, and the difference between groups was tested with the chi-square or Fisher exact test. The difference was statistically significant when a two-sided *P*-value was below 0.05.

## Results

The study included 505 children with a mean age of 3.8 ± 1.4 years, ranging between 1.02 to 7.4 years, with a male: female ratio of 1.05:1. The characteristics of the screened children, and history of pregnancy, delivery, neonatal period, infancy are presented in Table 1. Among our study group, 494 (97.8%) were residents in the district of the hospital, 7 (0.1%) in other capital districts, and 4 (0.8%) from lower Egypt. Almost all participants lived in urban communities [504 (99.8%)], apart from one living in rural areas. Parents declined any concerns regarding their children's growth or development except for 3 (0.6%) families who described global developmental delay of their children. Medical history was insignificant in 493 (97.6%) children, while 12 (2.4%) had positive history as follows; two children (0.4%) had glucose 6 phosphate dehydrogenase deficiency not presenting in acute hemolytic crisis, two (0.4%) had bronchial asthma, and each was positive in one child (0.2%), small for age as a newborn, Erb's palsy since birth, febrile convulsions, abnormal EEG treated with valproic acid, occasional undiagnosed syncopal attack, pica with constipation, and nocturnal enuresis. As regards the family history of the study group, 106 (21%) children were born to a consanguineous parent. Among children who had other siblings [262 (51.9%)], the number of siblings ranged between one to five with a median (IQR) of 1 (1–2) children, and 3 (0.6%) children were twins. Regarding their siblings, three (0.6%) brothers had G6PD, and each was reported in one (0.2%) brother; down syndrome, cardiac condition, EEG changes, asthma, febrile convulsions, and a brother died at four months of age with achondroplasia.

**Table 1:** Children characteristics and  history of the study group (*n* = 505)

**Variable**	**Values**
*Age in years; mean* ± *SD [min–max]*	3.8 ± 1.4 [1.02 – 7]
*Categories; N (%): Up to 5 years*	391 (77.4)
*Above 5 years*	114 (22.6)
*Gender; N (%): Male*	259 (51.3)
*Female*	246 (48.7)
**History of neonatal period and infancy**	
Physiologic jaundice;* N (%):*	140 (27.7)
Neonatal intensive care "NICU" admission; N (%):	98 (19.4)
Initial cause of admission:	
- *Respiratory distress*	57 (58.2)
- *Jaundice*	40 (40.8)
- *Hypoglycemia*	1 (1)
Duration of stay in days; median (IQR) [min–max]	5.5 (4 -10) [1 – 60]
Oxygen administration more than 4 weeks; N (%)	3 (0.6)
Blood transfusion at NICU	1 (0.2)
Type of feeding; N (%):	
- *Exclusive Breast On demand*	337 6.7)
- *Exclusive Breast then formula*	2 (0.4)
- *Breast & formula*	75 (14.9)
- *Breast & Animal*	3 (0.6)
- *Formula*	88 (17.4)
Exclusive breast feeding duration in months (*n* = 339): mean ± SD [min–max]	5.9 ± 1.97 [1–12]
Total duration of breast and/or bottle feeding in months; median (IQR) [min–max]	6 (5—7) [1.5 – 24]
Number of daily bottles (*n* = 168); N (%)	
- *Once or twice*	34 (20.2)
- *From two to 5*	71 (42.3)
- *More than 5*	63 (37.5)
Taken all vaccination; N (%)	491 (97.2)
Missed vaccination; N (%)	14 (2.8)
- *Not vaccinated at all*	4 (0.8)
- *Missed 4 months*	1 (0.2)
- *Missed 9 months*	1 (0.2)
- *Missed 18 months*	8 (1.6)
**History of pregnancy and delivery**	
Maternal infection; N (%):	
- *Undiagnosed infection*	2 (0.4)
- *Hepatitis C viral infection*	3 (0.6)
- *Herpes simplex viral infection*	1 (0.2)
Maternal disease; N (%):	
- *Hypertension "HTN"* ± *preeclampsia*	16 (3.2)
- *Gestational Diabetes Millitus "DM"*	6 (1.2)
- *DM & HTN*	5 (1)
- *Hypothyroidism*	3 (0.6)
- *Renal stone*	1 (0.2)
- Urinary tract infection "*UTI"*	1 (0.2)
Maternal treatment; N (%):	
- *Antihypertensive*	16 (3.2)
- *Insulin*	6 (1.2)
- *Insulin & anti-hypertensive*	5 (1)
- *Thyroxin*	3 (0.6)
- *Antibiotics*	2 (0.4)
- *Anticoagulant*	1 (0.2)
**Regular maternal intake of supplements (vitamins & iron);* N (%):***	158 (31.3)
Mode of delivery; N (%):	
- *Vaginal*	*161 (31.9)*
- ﻿Cesarian section "*CS"*	*344 (68.1)*
Eventful delivery; N (%):	
- *Premature rupture membranes*	5 (1)
- *Breech presentation*	3 (0.6)
- *Blood transfusion after post-partum bleeding*	2 (0.4)
- *Difficulty due to presence of uterine tumor*	1 (0.2)

Regarding the study group's examination, they were afebrile with no significant findings during chest, cardiac, and abdominal examinations. None had edema, clubbing, acanthosis, purpura, xanthoma, or jaundice. Three children had remarkable findings, each in one (0.2%) child, left leg burn scar, café au lait patches with a positive family history of neurofibromatosis, and facial hypopigmentation. The anthropometric measures, examination findings, and results of the complete blood count are shown in Table 2.

**Table 2:** Anthropometry, examination, and laboratory findings of the study group (*n* = 505)

**Variable**	**Values**
**Anthropometric measures**	
Weight in Kg; mean ± SD [min–max]	15.5 ± 4.02 [6 – 48]
Z score of weight; median (IQR)	-0.28 (-0.95 – 0.5) [-6.6 – 3.3]
Weight percentiles; median (IQR)	39 (17.3 – 68.3) [0 – 100]
Height in cm; mean ± SD	97.5 ± 11.02 [71 – 136]
Z score of height; median (IQR)	-0.5 (-1.2 – 0.2) [-5.4 – 3.8]
Height percentiles; median (IQR)	30.2 (12.3 – 58.1) [0 – 100]
BMI; mean ± SD	16.1 ± 2 [9.1 – 30.2]
Z score of BMI; median (IQR)	0.2 (-0.6 – 0.99) [-7.7 – 3.9]
BMI percentiles; median (IQR)	56.7 (27.95 – 84) [0 – 100]
Q95 (> 5 years); mean ± SD	18.3 ± 0.4 [17.8 – 20.1]
BMI p95; mean ± SD	0.9 ± 0.1 [0.6 – 1.6]
**Examination findings**	
Signs of vitamins deficiencies; N (%):	
- *Pallor*	119 (23.6)
- *Signs of rickets*	10 (2)
- *Dermatitis*	8 (1.6)
- *Acro-orificial rash*	5 (1)
- *Mucositis & chelitis*	3 (0.6)
- *Alopecia areata*	2 (0.4)
- *Spooning of the nail*	1 (0.2)
**Laboratory findings**	
Total leukocytic count "*TLC" (*× *10*^*3*^*/cmm); Mean* ± *SD [min–max]*	8.2 ± 2.9 [2.64 – 21.2]
Hemoglobin *"HB" (mg/dl); Mean* ± *SD [min–max]*	11.7 ± 1.1 [8.10 – 14.6]
Platelet "*PLT" (*× *10*^*3*^*/ul); Mean* ± *SD [min–max]*	382.15 ± 105 [101 – 750]
Mean corpuscular volume *"MCV" in fl; Mean* ± *SD [min–max]*	73 ± 6.1 [56.9 – 84.6]

The screening results were average in 228 (45.1%) children, while 277 (54.9%) had abnormal anthropometry, anemia, or both. The frequencies of abnormal anthropometry & anemia are shown in Table 3. Children with stunting were ten girls and five boys, and those with overnutrition were 63 (57.2%) boys and 62 (42.8%) girls.

**Table 3:** The frequency of abnormal anthropometry and anemia in the study group (*N* = 505)

	**Screened group** ***N* = 505**	** Age classification**		
		**Up to 5 years (*n* = 391)**	**At or above 5 years (*n* = 114)**	***P* value**
Gender; N (%):				
- *Male*	259 (51.3)	203 (51.9)	56 (49.1)	0.7
- *Female*	246 (48.7)	188 (48.1)	58 (50.9)	
Overall screening results; N (%):				
- *Normal*	228 (45.1)	175 (44.8)	53 (46.5)	0.7
- *Abnormal*	277 (54.9)	216 (55.2)	61 (53.5)	
Anthropometric measures; N (%):				
- *Normal*	319 (63.2)	252 (64.5)	80 (70.2)	0.3
- *Abnormal*	186 (36.8)	139 (35.5)	34 (29.8)	
Abnormal anthropometry; N (%):				
*Undernutrition:*	48 (9.5)	38 (9.7)	10 (8.8)	0.7
- *Underweight*	◦ 33 (6.6)	◦ 27 (6.9)	◦ 6 (5.3)	◦ 0.7
- *Wasting*	◦ 33 (6.6)	◦ 28 (7.2)	◦ 5 (4.4)	◦ 0.4
- *Stunting*	◦ 15 (3)	◦ 10 (2.6)	◦ 5 (4.4)	◦ 0.3
*Over-nutrition*	125 (24.8)	101 (25.8)	24 (21.1)	0.3
- *Overweight*	◦ 43 (8.5)	◦ 26 (6.6)	◦ 17 (14.9)	◦ < 0.001*
- *Obesity*	◦ 12 (2.4)	◦ 5 (1.3)	◦ 7 (6.1)	◦ < 0.001*
- *Possible high abnormality*	◦ 70 (13.9)	◦ 70 (17.9)	◦ 0	◦ < 0.001*
*Short stature*	29 (5.7)	27 (6.9)	2 (1.8)	0.04*
- *With normal BMI*	◦ 13 (2.6)	◦ 13 (3.3)	◦ 0	◦ 0.08
- *With overweight/obesity*	◦ 6 (1.2)	◦ 4 (1)	◦ 2 (1.8)	◦ 0.6
- *With possible overweight*	◦ 10 (1.98)	◦ 10 (2.6)	◦ 0	◦ 0.1
Presence of Anemia; N (%):	141 (27.9)	100 (25.6)	41 (36)	0.03*
Having both anemia & abnormal anthropometry; N (%):	50 (9.9)	36 (9.2)	14 (12.3)	0.4

*P< .05

Anemia was diagnosed in 141 (27.9%) children in the study group. Cases with anemia had a mean hemoglobin of 10.5 ± 0.7 mg/dl, ranging between (8.1–11.4). About 65% of cases with anemia had a low MCV and or RDW > 15. Cases with anemia were significantly older than those with normal hemoglobin. The comparison of demographic data & anthropometry in participants with anemia and those without anemia is shown in Table 4. The child who received valproate had average anthropometry (~ 50th percentile), and anemia with low MCV and high RDW.

**Table 4:** Comparison of demographic data and anthropometry in the study group categorized by the normal hemoglobin level (*N* = 505)

	**Normal Hemoglobin (*N* = 364)**	**Anemia (*N* = 141)**	***P* Value**
Age; mean ± SD	3.6 ± 1.4	4.1 ± 1.3	< 0.001*
• *Below 4 years*	291 (79.9)	100 (70.9)	0.03*
• *Below 4 years*	227 (62.4)	58 (41.1)	< 0.001*
Gender; N (%):			
• *Male*	189 (51.9)	70 (49.6)	0.6
• *Female*	175 (48.1)	71 (50.4)	
Normal anthropometry; N (%):	228 (62.6)	91 (64.5)	0.8
Abnormal anthropometry; N (%):			
• *Undernutrition:*	40 (11)	8 (5.7)	0.09
◦ *Underweight*	◦ 29 (72.5)	◦ 4 (50)	0.04*
◦ *Wasting*	◦ 27 (67.5)	◦ 6 (75)	0.2
◦ *Stunting*	◦ 14 (35)	◦ 1 (12.5)	0.08
• *Over*-nutrition	86 (23.6)	39 (27.7)	0.4
◦ *Overweight / Obesity*	◦ 34 (39.5)	◦ 21 (53.8)	0.08
◦ *Possible high abnormality*	◦ 52 (60.5)	◦ 18 (46.2)	0.7
• *Short stature*	20 (5.5)	9 (6.4)	0.8
◦ *With normal BMI*	◦ 10 (50)	◦ 3 (33.3)	0.8
◦ *With overweight/obesity*	◦ 3 (15)	◦ 3 (33.3)	0.4
◦ *With possible overweight*	◦ 7 (35)	◦ 3 (33.3)	0.9

*P< .05

**Fig. 1:**
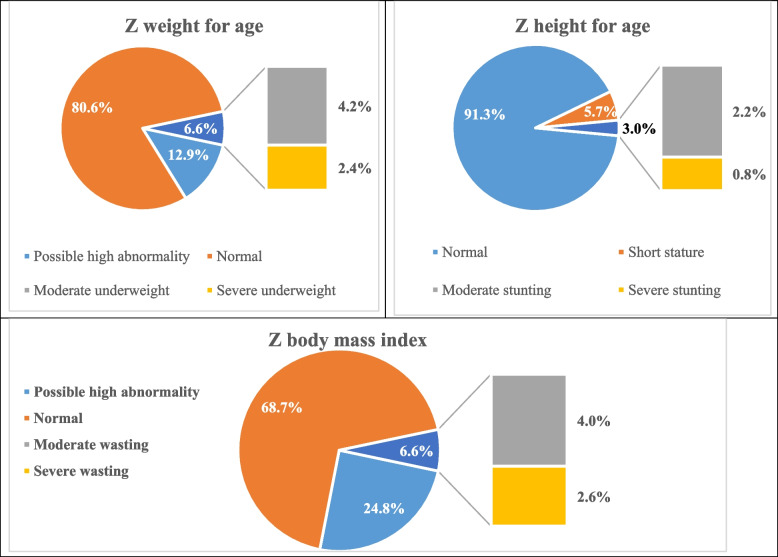
The distribution of the Z score for weight, height, and body mass index in the study group (*n* = 505)

**Fig. 2:**
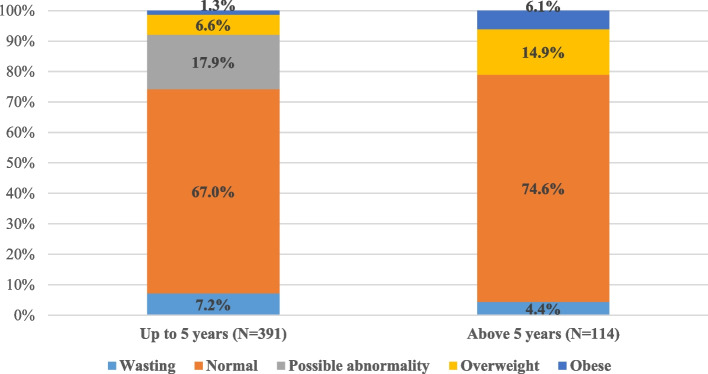
Classification of body mass index Z score of the study group classified by the age of 5 years; showing wasting and obesity rates (*n* = 505)

We used the Z score of weight, height, and BMI to classify the nutritional status of the study group, showing the normal and the abnormal status, Fig. 1. We classified the group at five and presented the body mass index Z score to show the frequency of normal, under-, and over-nutrition status, Fig. 2. In those under five years, 70 (17.9%) had a z score between 1 & 2, "possible abnormality," not qualifying the definition of overweight/ obesity.

## Discussion

Our screening project included 505 Egyptian children below the eligible school age during recruitment. Their ages ranged between one year and 7.4 years, with a mean age of 3.8 ± 1.4 years, with almost equal gender distribution. All participants, except one, were living in an urban community. We used anthropometric measures and complete blood count to screen the group for malnutrition and anemia. Egypt is an African country located in the Middle East and is a lower-middle-income country. Screening children early in life is mandatory to detect nutritional problems early and take proper action to solve them. Preschools represent a critical period for subsequent health in life, where nutrition and care are essential in determining their ongoing and future quality of life.

From our results, 48 (9.5%) children had undernutrition. Among children with undernutrition (9.5%), 33 (6.6%) were underweight, 33 (6.6%) wasted, and 15 (3%) were stunted, with no significant difference between the frequencies in children below or above the age of five years. Our observed undernutrition rates are lower than those published in 2014 via a national survey, which reported underweight in 11.3%, wasting in 5.7%, and stunting in 37.5%. The national study addressed a significant burden of undernutrition in children aged 18 months to 5 years, particularly for stunted growth [10]. Also, ours are lower than preschools in the Eastern Mediterranean region, where estimated rates for stunting, wasting, and being underweight were 28%, 8.69%, and 18%, respectively [2]. As a leading cause of mortality in 45% of preschoolers, particularly in low-income and middle-income countries, detecting children with undernutrition is vital in promoting their health [1].

In our study, 33 (6.6%) were underweight; 4.2% had moderate form, while 2.4% had severe form. Being underweight is a form of undernutrition, and cases may be stunted, wasted, or both [1].

We observed wasting in 6.6%, where 4% had moderate & 2.6% had severe form. Our observed wasting rate is similar to the global rate reported among preschools in 2020, which was 6.7% [11]. Wasting is an indication of a recent & marked weight loss. It is mainly a result of nutritional deficiencies or infections like diarrhea or both. Moderate and severe wasting is considered a leading cause of death if untreated properly [1]*.*

We found stunting in 15 (3%) children, with a female: male ratio of 2:1. Of them, 2.2% had moderate stunting, and 0.8% had severe form. Stunting is usually a reflection of chronic or recurrent undernutrition, and it holds children back from reaching their expected physical and cognitive potential [1]*.* Besides, stunting is associated with an outstanding liability for getting infections, which suggests the possibility of an associated immune dysfunction [12]*.* Unlike our findings, previous studies observed higher rates of stunting among boys [13], with some showing significance [14].

According to our methodology, we detected stunting in 3% of screened young children. If we consider all children with height for age Z score below -2 SD regardless of the presence of underweight, 44 (8.7%) children will be labeled as stunted. In either case, our observed prevalence of stunting qualifies for a low degree of severity as a public health problem, per the WHO classification for stunting [7]*. Besides,* our rate is much lower than the global prevalence in children under five years, estimated at 22% in 2022 [11]***. ***Stunting growth rates vary significantly between countries, ranging from 1.3% to 55.9%; it is the most common form of malnutrition in children worldwide. It is a significant health priority in children globally, indicating linear growth failure. As increased height is a sign of overall children's health, stunting reflects inequality in development and below-average health due to inadequate care and nutrition [15].

From our results, children with a height for age Z score below -2 SD and not suffering from being underweight were considered short. A total of 29 (5.7%) children had short stature; out of them, 16 (55.2%) children had above accepted BMI Z score, and 13 (44.8%) had short stature with normal BMI.

From our observations, children with overnutrition were 125 (24.8%), 43 (8.5%) of them were overweight, 12 (2.4%) were obese, and 70 (13.9%) had high BMI Z scores that did not qualify the definition of overweight. Therefore, 55 (10.9%) were overweight or obese, and 13.9 were above normal limits according to the definition. The prevalence of over-nutrition was significantly higher in children above five, with comparable gender distribution. Our observed over-nutrition rates are much higher than the current global prevalence of overweight in preschool ages, estimated at 5.7% [10]*.* Also, it is higher than the estimated prevalence of over-nutrition in the Eastern Mediterranean region preschoolers, which was 8.42% [2]. In the past decades, over-nutrition and its complications have been described in adolescents and older children. With the continuing obesity pandemic, many preschools are defined as overweight or obese [16]*. *Unlike our observations, the male gender was associated with a higher prevalence of being overweight than females in preschool children [14]*.* 

Our current prevalence of overnutrition is higher than that published by a national survey in 2014, which reported a prevalence of 14.9% in children below five years. The same survey reported a 21.3% prevalence for overweight and obesity among children aged 13–15 years [overweight: 16.46% & obesity: 4.83%] [10]. The observed increment trend of over-nutrition in young children in Egypt follows the augmentation of obesity rates worldwide. Earlier studies had observed doubling rates of childhood obesity between 1990 & 2015 worldwide, from 4.2% to 7.8% [17]*.*

In our work, children with undernutrition and those with overnutrition coexist in the studied young children. Earlier studies have reported the coexistence of both forms of malnutrition in preschool children within the same communities [18, 19] and particularly in the Middle East [20]*.*

Regarding our national situation, the underweight rates declined, while that of over-nutrition increased, reaching 2.5 times that of undernutrition. Previous studies have reported different associations between undernutrition and overnutrition. In agreement with our results, studies have observed an upward trend for overnutrition associated with declining undernutrition in young preschoolers [21–23]*.* Contrary, some reports observed high rates of both over-nutrition and undernutrition in preschoolers have been reported in some low- and middle-income communities, indicating a double burden of malnutrition [1, 21, 24].

Anemia was diagnosed in 141 (27.9%) children in the study group, and it was significantly more frequent among children at or above the age of five without significant gender discrimination. Our observed anemia prevalence qualifies as a moderate public health problem per the WHO classification, i.e., between 20%-39%. Similarly, an earlier national survey found that 27.2% of preschool children were anemic [10]. Preschool children represent a risk group for developing anemia [25]. Children living in the Eastern Mediterranean region have considerably high rates of anemia, at about 49%. Anemia is a medical condition reflecting the overall child's health in many aspects, and its presence carries a risk of subnormal cognitive functions and developmental skills [1, 26].

In our study, the frequency of abnormal anthropometry was comparable between children with anemia and those with normal hemoglobin, except for underweight which was significantly more common among children with normal hemoglobin. Contrarily, studies have observed an association between being undernourished and anemia in children, particularly preschoolers. Woldie and coworkers [27] found that undernourished children were usually anemic due to micronutrient deficiency in their diets. Also, below-average hemoglobin levels can negatively affect linear growth. Besides, *Luo and colleagues* [28] noticed that anemic children were shorter for their age standards, and a higher percentage of them were stunted.

In our sample, about half participants had one form of malnutrition or anemia or both, with no significant difference in children below or above the age of five years. Abnormal anthropometry was observed in 36.8% of children, anemia in 27.9%, and 9.9% had anemia and malnutrition. The high rate of positive screening reflects an alarming sign of the triple burden of malnutrition in our study group. Our observations follow previous reports emphasizing that superadded negative effect of micronutrient deficiency—mainly iron deficiency anemia- to malnutrition creates a triple burden in communities where all patterns are well represented [29]. Nutritional inadequacy in early life can induce malnutrition, either undernutrition or over-nutrition, micronutrient deficiency, or a combination. The drawbacks of nutritional deficiencies in early life included some irreversible cognitive dysfunctions, below-expected school performance, and growth affection and its sequel [30, 31]*. *Literature has postulated common unhealthy dietary patterns for anemia and forms of malnutrition in children, i.e., low nutritional values (protein and micronutrients) and energy-dense contents at a lower price [1, 32].

Limitations of our work included the cross nature of screening, which cannot identify the etiology of malnutrition or anemia in our study group. Also, the socioeconomic status which greatly affects the nutrition status was not assessed in our work, and its effect could not be weighted. We expected more children to be screened, but the families were not motivated to bring their children to the hospital following the maximum era of Coronavirus infections, where the hospital was selected as an isolation center for COVID-19 patients.

### Conduction site

Helwan University hospital at Badr city, Egypt. Data presented are the screening results and follow-up data are still pending.

## Conclusions

From the presented data, we concluded that malnutrition and anemia in preschools are still a heavy burden, with a shift of the weight of malnutrition towards the overnutrition side. As a national observation, an upward trend for overweight & obesity was associated with a declining one for undernutrition in preschoolers. The added burden of anemia exaggerates the problem of malnutrition in our communities with limited resources.

We recommend similar screening studies in preschools in different districts to identify the problem and find suitable solutions for each community. Early recognition of malnutrition should be a primary concern in the medical services for preschools, having tremendous complications. Malnutrition negatively affects young children's health, being a leading cause of morbidity and mortality with long-term economic and social impact and impairment of their cognitive functions, learning abilities, and creativity in their future life.

## Data Availability

The datasets used and/or analyzed during the current study available from the corresponding author on reasonable request.
